# A Case of Ileocecal IgG4-Related Sclerosing Mesenteritis Diagnosed by Endoscopic Ultrasound-Guided Fine Needle Aspiration using Forward-Viewing Linear Echoendoscope

**DOI:** 10.1155/2019/2530487

**Published:** 2019-10-27

**Authors:** Yuichi Takano, Fumitaka Niiya, Takahiro Kobayashi, Eiichi Yamamura, Naotaka Maruoka, Tomoko Norose, Nobuyuki Ohike, Masatsugu Nagahama

**Affiliations:** ^1^Division of Gastroenterology, Department of Internal Medicine, Showa University Fujigaoka Hospital, Yokohama, Kanagawa, Japan; ^2^Department of Diagnostic Pathology, Showa University Fujigaoka Hospital, Yokohama, Kanagawa, Japan

## Abstract

A 25-year-old woman had undergone removal of a cryptogenic tumor in the left maxillary sinus 1 year prior to presentation. The patient experienced abdominal pain for 4 days with repeated vomiting episodes; therefore, she was transferred to our hospital by an ambulance. Contrast-enhanced computed tomography revealed a 3-cm tumor in the ileocecal region, which caused small bowel obstruction. Contrast imaging of the ileus tube showed extrinsic compression of the ileocecal region. Forward-viewing linear echoendoscope revealed an irregular hypoechoic tumor measuring 3 cm outside the gastrointestinal tract. Using a 25G needle, endoscopic ultrasound-guided fine needle aspiration (EUS–FNA) was performed. Pathological finding was an inflammatory fibrous tissue with diffuse lymphoplasmacytic infiltration, with more than 10 IgG4-positive cells detected in a high-power field. Re-examination of a pathology specimen of the maxillary sinus tumor provided by the previous attending physician revealed that the inflammatory tissue had diffuse lymphoplasmacytic infiltration, which were accompanied by storiform fibrosis and obliterative phlebitis. Immunostaining revealed more than 50 IgG4-positive cells in a high-power field, a finding suggestive of IgG4-related disease. The serum IgG4 level was 21 mg/dl, which was within the normal range. Treatment was initiated with prednisolone at a dose of 50 mg/day, and the dose was later tapered off. CT and MRI performed 2 months later showed complete disappearance of the ileocecal tumor. The final diagnosis was asynchronously occurring IgG4-related maxillary sinusitis and sclerosing mesenteritis.

## 1. Introduction

IgG4-related disease (IgG4-RD) is an idiopathic inflammatory disorder that affects organs throughout the body [1]. It is characterized by swelling of the affected organs, increase in serum IgG4 level, peculiar histological findings (diffuse lymphoplasmacytic infiltration, storiform fibrosis, obliterative phlebitis, and numerous IgG4-positive cells), and good response to steroid treatment. The most commonly affected organs include the pancreas, parotid glands, lacrimal glands, retroperitoneum (around the aorta), and kidneys, with occurrence in the gastrointestinal tract, spleen, and brain (excluding the pituitary gland) considered rare [2].

Sclerosing mesenteritis is also an inflammatory disease of unknown origin [3]. In recent years, few cases of sclerosing mesenteritis possibly associated with IgG4-RD have been reported [3–12]. These cases were diagnosed by surgical resection or surgical biopsy (laparoscopic biopsy), with no reports available on cases diagnosed by endoscopic ultrasound-guided fine needle aspiration (EUS-FNA). In this study, we report a case of ileocecal IgG4-related sclerosing mesenteritis diagnosed by EUS-FNA using forward-viewing linear echoendoscope. Because the patient showed good response to steroid treatment, surgery could be avoided.

## 2. Case Presentation

A 25-year-old woman had undergone tumor removal in the left maxillary sinus at the Otolaryngology Department of another hospital 1 year prior to presentation. Pathological diagnosis was inflammatory pseudotumor of unknown origin. The patient had no remarkable family history and did not consume alcohol or smoke. Owing to intermittent abdominal pain for 4 days and frequent vomiting in the morning of the examination day, the patient was transported by ambulance. On arrival, her vital signs were as follows: body temperature, 36.1°C; blood pressure, 115/85 mmHg; and heart rate, 86 beats/min. Physical examination revealed severe tenderness in the right lower abdomen. There were no signs of rebound pain or muscle guarding. Blood test showed WBC count and CRP level of 16740/*μ*l and 10.33 mg/dl, respectively, indicating increased inflammatory response. Further, biochemical measurements were as follows: IgA, 215 mg/dl; IgM, 175 mg/dl; IgE, 246 IU/ml; IgG, 1275 mg/dl; IgG4, 21 mg/dl; ANA, negative; ds-DNA Ab, negative; PR3-ANCA, negative; and MPO-ANCA, negative. No findings were suggestive of an autoimmune disease or vasculitis. The serum IgG4 level was within the normal range. Tumor marker levels were as follows: sIL-2R, 611 U/ml; CA19-9, 9.6 U/ml; and CEA, 2.4 ng/ml; a slight increase in sIL-2R level was observed.

**Figure 1:**
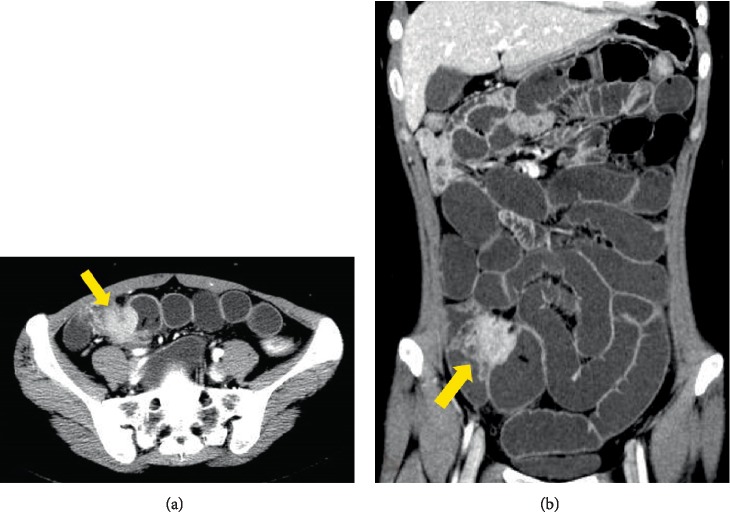
(a), (b) CECT showing a 3-cm hypervascular tumor (arrow) in the ileocecal region accompanied by small bowel obstruction.

**Figure 2:**
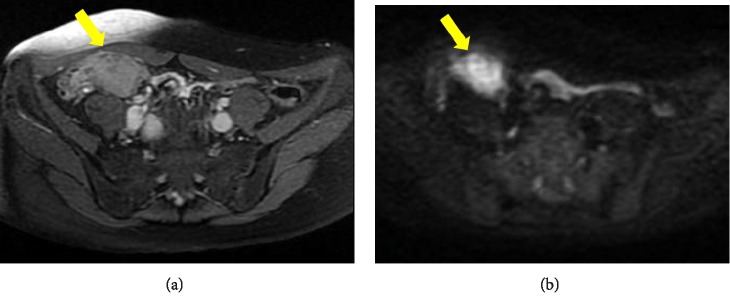
(a) MRI T2-weighted images showing mild high intensity lesions in the ileocecal region (arrow). (b) MRI diffusion-weighted images showing decreased diffusion in lesions (arrow).

**Figure 3:**
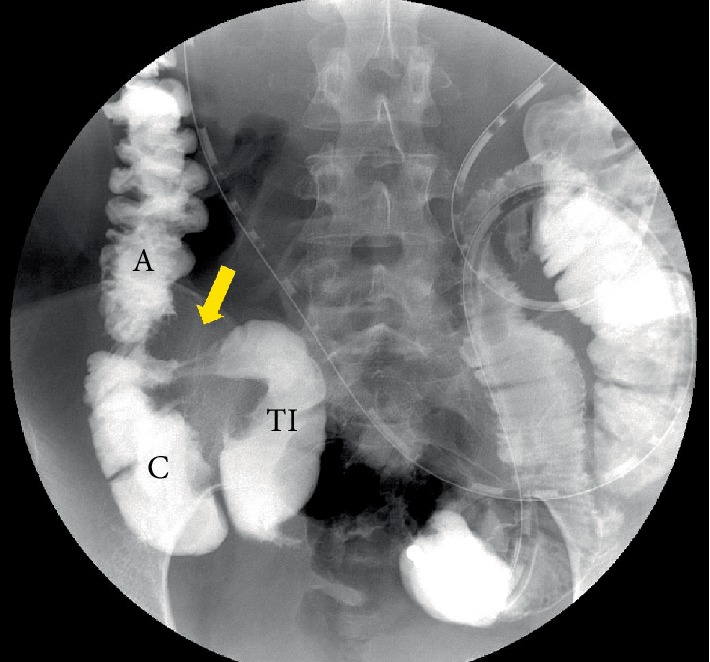
Contrast imaging of the ileus tube showing extrinsic compression of the ileocecal region (arrow). TI: terminal ileum, C: cecum, A: Ascending colon.

**Figure 4:**
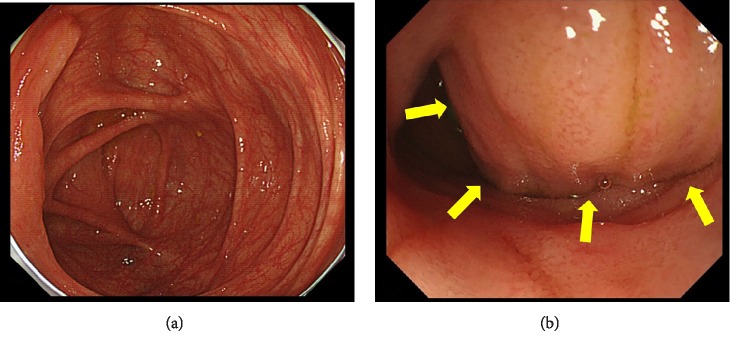
(a) Endoscopy of the lower gastrointestinal tract showing no abnormalities in the mucous membrane of the cecum and ileum. (b) Extramural compression was detected in the terminal ileum (arrow).

Contrast-enhanced computed tomography (CECT) showed a 3-cm hypervascular tumor in the ileocecal region, revealing small bowel obstruction (Figure 1). The lesion emitted slightly high intensity on simple magnetic resonance imaging (MRI) T2-weighted images and showed decreased diffusion on diffusion-weighted images (DWI) (Figure 2). Accordingly, an ileus tube was rapidly placed after hospitalization. Contrast imaging of the ileus tube showed extrinsic compression of the ileocecal region (Figure 3). While endoscopy of the lower gastrointestinal tract revealed no abnormalities in the mucous membrane of the ileum and cecum, the terminal ileum exhibited extramural compression (Figure 4).

Based on the findings, an extraintestinal tumor of the ileocecal region and small bowel obstruction were diagnosed. Differential diagnoses included gastrointestinal stromal tumor (GIST), schwannoma, malignant lymphoma, adenocarcinoma, and inflammatory pseudotumor. Although we considered performing ileocecal resection, we decided to first implement preoperative histological examination after consultation with the surgical department.

**Figure 5:**
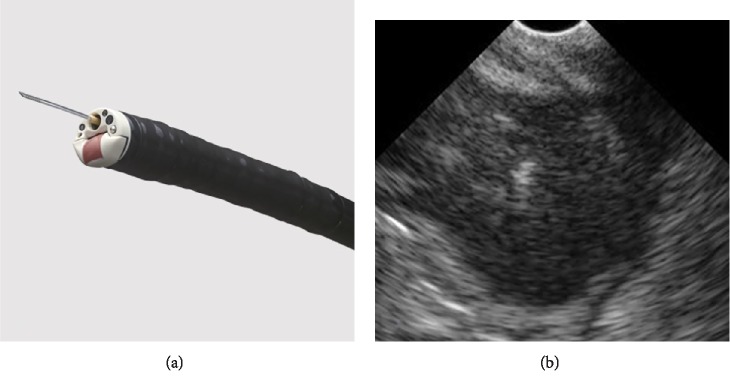
(a) Forward-viewing linear echoendoscope, TGF-UC180J (Olympus Medical Systems Corp, Tokyo, Japan). (b) EUS revealed an irregular hypoechoic tumor measuring 3 cm outside the gastrointestinal tract.

A forward-viewing linear echoendoscope (TGF-UC180J, Olympus Medical Systems Corp, Tokyo, Japan) was inserted into the cecum, which revealed a 3-cm hypoechoic tumor with clear boundaries and irregular periphery outside the gastrointestinal tract (Figure 5).

**Figure 6:**
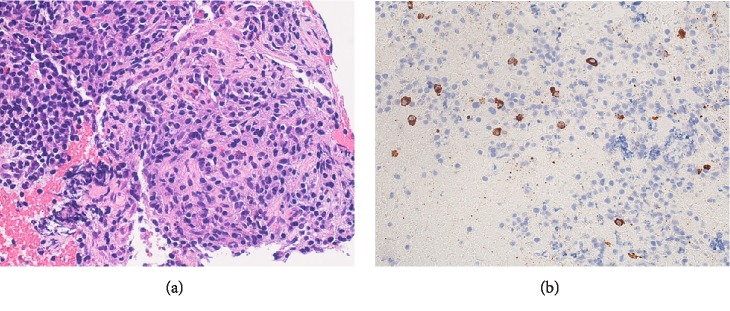
(a) A specimen obtained by EUS-FNA. Pathologically, the lesion was found to be composed of inflammatory fibrous tissues with lymphoplasmacytic infiltration (Hematoxylin-Eosin Stain, x400). (b) IgG4 staining revealed more than 10 IgG4-positive cells in an HPF (IgG4 Stain x400).

Using a 25G needle (Expect™ SlimLine, Boston Scientific Japan, Tokyo, Japan), two sessions of EUS-FNA were performed (Supplementary Video [Supplementary-material supplementary-material-1]). The procedure was completed without any complications. Pathologically, the lesion was found to comprise inflammatory fibrous tissues with lymphoplasmacytic infiltration, and more than 10 IgG4-positive cells were detected in a high-power field (HPF) (Figure 6). While IgG staining was difficult because it involved co-staining, the IgG4/IgG ratio exceeded 40%.

**Figure 7:**
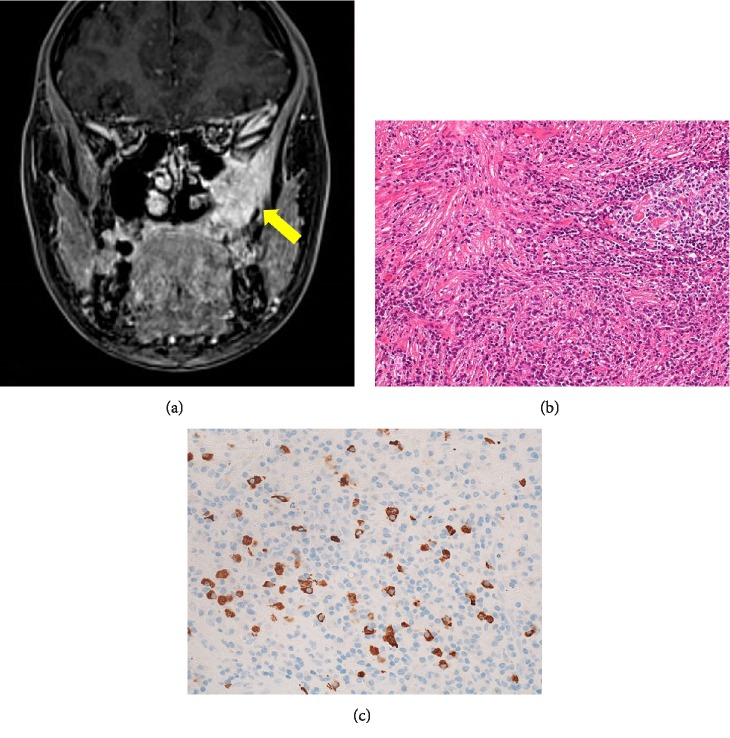
(a) Contrast-enhanced MRI showing an irregular tumor in the left maxillary sinus (arrow). (b) Pathologically, diffuse lymphoplasmacytic infiltration and storiform fibrosis are observed (Hematoxylin-Eosin Stain, x200). (c) More than 50 IgG4-positive cells are observed in an HPF (IgG4 Stain, x400).

Images and pathological specimens of the maxillary sinus tumor provided by the previous attending doctor were re-evaluated. Contrast-enhanced MRI revealed an irregular tumor showing contrast in the left maxillary sinus (Figure 7).

Histologically, the lesion was an inflammatory tissue containing diffuse lymphoplasmacytic infiltration accompanied by storiform fibrosis and obliterative phlebitis. Immunostaining revealed more than 50 IgG4-positive cells in an HPF, which was consistent with IgG4-RD (Figure 7). IgG staining was difficult because it involved co-staining. There were no abnormal findings in other organs throughout the body (lacrimal salivary gland, mediastinal and hilar lymph node, lung, pancreas, bile duct, kidney, artery).

**Figure 8:**
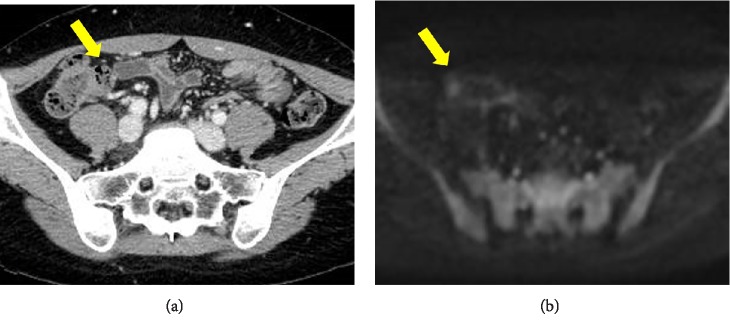
(a) CECT showing complete disappearance of the tumor at 2 months after steroid treatment (arrow). (b) MRI diffusion-weighted images indicate the absence of any lesion (arrow).

The patient was diagnosed with IgG4-related maxillary sinusitis and sclerosing mesenteritis. The patient weighed 52 kg and prednisolone was started at a dose of 50 mg as 1 mg/kg, which was later tapered off. CT and MRI performed 2 months later showed complete disappearance of the ileocecal tumor (Figure 8).

The patient received maintenance therapy with 5 mg/day prednisolone for 1 year thereafter, and IgG4-RD recurrence was not observed.

## 3. Discussion

In 2001, Hamano et al. reported that serum IgG4 is a marker specific to autoimmune pancreatitis [13]. They later demonstrated IgG4-positive cell infiltration in the pancreatic tissue [14]. Another study discovered an inflammatory fibrous disease characterized by increased serum IgG4 level and IgG4-positive cell infiltration in general organs, thereby establishing a novel concept of IgG4-RD [1, 15].

In recent years, few cases of sclerosing mesenteritis possibly associated with IgG4 have been reported. A PubMed search of studies published in 2000–2019 was conducted using the keywords “IgG4” and “sclerosing mesenteritis,” and a total of 17 cases (those with ≥10 IgG4-positive cells, excluding reports with fewer or an unspecified number of cells) were found [3–12].

We examined a total of 18 cases including our case (Table 1). The mean age of the patients was 58 years (range: 25–82 years), male: female ratio was 12:6, and mean serum IgG4 level was 95 mg/dl (range: 21–171 mg/dl). Further, the mean IgG4-positive cell count in an HPF was 63 (range: 10–100). Two cases complicated other organ involvements, and the breakdown was retroperitoneal fibrosis and maxillary sinusitis. Regarding diagnostic method, 15 cases underwent surgical resection and 2 underwent laparoscopic biopsy. Only our case was diagnosed with EUS-FNA. None of the cases received steroids except our case. For the 2 cases diagnosed by laparoscopic biopsy, clinical progress was followed up without treatment, and the tumors eventually showed reduction in size [8].

In conclusion, this condition is common among middle-aged men. The serum IgG4 level tends to be within the normal range or slightly increased, and other organ involvements are uncommon. Approximately 90% of the cases were diagnosed by surgical resection, which suggests the difficulty in preoperative diagnosis. Although the first option for the treatment of IgG4-RD is steroid, no cases were treated with steroids other than our case.

To date, no cases have been diagnosed using EUS-FNA, possibly because lesions tend to be present in deep parts of the gastrointestinal tract, making paracentesis difficult. At present, oblique-viewing linear echoendoscope is usually employed for EUS-FNA. However, in the present case, EUS-FNA was performed from the cecum. Because inserting an oblique-viewing echoendoscope into the cecum involved the risk of intestinal damage and perforation, a forward-viewing echoendoscope was used instead. The puncture was from the lower gastrointestinal tract and there was a risk of perforation and peritonitis. Therefore, EUS-FNA using a thin needle (25G) was performed. The procedure was performed without complications. Even with a 25G needle, enough specimens were obtained, and immunostaining could be performed easily.

In many cases, preoperative diagnoses of malignant diseases, such as GIST, malignant lymphoma, and cancer, are done based on imaging, resulting in surgeries being performed without biopsies. However, in the case of IgG4-RD, steroid is usually effective in shrinking lesions [1]. As such, performing preoperative biopsies is critical. Unnecessary surgery should be avoided as much as possible, particularly in young patients. In the present case, the lesion disappeared after steroid treatment, and small bowel obstruction improved without surgery. Therefore, EUS-FNA proved to be highly beneficial for the patient.

While the serum IgG4 level in our patient was within the normal range, not all patients with IgG4-RD exhibit increased serum IgG4 levels. In fact, an international study revealed that among patients who are histologically diagnosed with type 1 autoimmune pancreatitis (a pancreatic manifestation of IgG4-RD), 37% had normal serum IgG4 levels [16]. In particular, an increase in the serum IgG4 level tends to be subdued in cases of IgG4-related sclerosing mesenteritis. Histological examination needs to be proactively performed rather than relying solely on serum IgG4 findings.

Recently, cases wherein the serum IgG4 level was normal and IgG4-positive cells were scarcely detected despite showing typical pathological findings of IgG4-RD (storiform fibrosis, obliterative phlebitis, and diffuse lymphoplasmacytic infiltration) have been reported [17–19]. Therefore, further research is warranted because IgG4 may not be a prerequisite for the pathology of the disease.

The elevation of WBC and CRP is not a typical findings in IgG4 related disease. The patient had a bowel obstruction due to the lesion, and it took several days to visit our hospital. Inflammation due to intestinal obstruction may have caused a mild increase in inflammatory response (WBC and CRP).

The diagnosis of IgG4-related sclerosing mesenteritis is still debatable. Avincsal et al. reported that although sclerosing mesenteritis shares histological characteristics with IgG4-RD, given the scarcity of cases with elevated serum IgG4 and other organ involvements, it is possible that many of them are not IgG4-related [8]. They concluded that the possibility of IgG4-RD resulting in lesions in the mesentery is extremely rare.

According to the pathological consensus regarding IgG4-RD published in 2012, a lesion needs to meet the following criteria (at least 3, ideally 4) to be considered an IgG4-RD in a new organ [20]: (1) characteristic pathological findings; (2) increase in serum IgG4 level; (3) good response to steroid treatment; and (4) IgG4-RD in other organs. The present case satisfied criteria 1, 3, and 4, which was considered adequate evidence to warrant the diagnosis of IgG4-related sclerosing mesenteritis.

We treated a case of IgG4-related maxillary sinusitis and sclerosing mesenteritis occurring asynchronously. Although the serum IgG4 level was within the normal range, histological findings and the patient's good response to steroid treatment were consistent with IgG4-RD. Although extremely rare, IgG4-related sclerosing mesenteritis does appear to exist. Biopsies need to be proactively performed to avoid unnecessary surgical interventions. Forward-viewing echoendoscope is useful for conducting biopsy from the deep gastrointestinal tract.

## Figures and Tables

**Table 1:** Literature cases.

Case	Age	Sex	Serum IgG4 (mg/dl)	IgG4 positive cells/HPF	Other organ involvement	Diagnosis method	Corticosreroid	Reference
1	48	F	NA	12	None	Resection	No	[3]
2	74	M	NA	>100	None	Resection	No	[3]
3	46	M	NA	>100	None	Resection	No	[3]
4	52	M	NA	13	None	Resection	No	[3]
5	63	F	NA	16	None	Resection	No	[3]
6	65	M	NA	16	None	Resection	No	[3]
7	82	F	171	130	None	Resection	No	[4]
8	42	M	119	60	None	Resection	No	[5]
9	53	M	127	75	None	Resection	No	[6]
10	64	M	81	38	Retroperitoneal fibrosis	Resection	No	[7]
11	76	M	63.5	56	None	Laparoscopic biopsy	No	[8]
12	77	M	43.2	46	None	Laparoscopic biopsy	No	[8]
13	54	M	105	85	None	Resection	No	[8]
14	77	F	114	253	None	Resection	No	[9]
15	56	F	164	33	None	Resection	No	[10]
16	25	M	90	>50	None	Resection	No	[11]
17	60	M	47	>40	None	Resection	No	[12]
Our case	25	F	21	>10	Maxillary sinusitis	EUS-FNA	Yes	

## Supplementary Materials

Supplementary Materials Video 1: EUS-FNA was performed using a 25G needle.
